# Identification of antigenic *Sarcoptes scabiei* proteins for use in a diagnostic test and of non-antigenic proteins that may be immunomodulatory

**DOI:** 10.1371/journal.pntd.0005669

**Published:** 2017-06-12

**Authors:** Marjorie S. Morgan, S. Dean Rider, Larry G. Arlian

**Affiliations:** Department of Biological Sciences, Wright State University, Dayton, OH, United States of America; University of California San Diego School of Medicine, UNITED STATES

## Abstract

**Background:**

Scabies, caused by the mite, *Sarcoptes scabiei*, infects millions of humans, and many wild and domestic mammals. Scabies mites burrow in the lower stratum corneum of the epidermis of the skin and are the source of substances that are antigenic or modulate aspects of the protective response of the host. Ordinary scabies is a difficult disease to diagnose.

**Objective:**

The goal of this project was to identify *S*. *scabiei* proteins that may be candidate antigens for use in a diagnostic test or may be used by the mite to modulate the host’s protective response.

**Methods:**

An aqueous extract of *S*. *scabiei* was separated by 2-dimensional electrophoresis and proteins were identified by mass spectrometry. A parallel immunoblot was probed with serum from patients with ordinary scabies to identify IgM and/or IgG-binding antigens. The genes coding for 23 selected proteins were cloned into *E*. *coli* and the expressed recombinant proteins were screened with serum from patients with confirmed ordinary scabies.

**Results:**

We identified 50 different proteins produced by *S*. *scabiei*, 34 of which were not previously identified, and determined that 66% were recognized by patient IgM and/or IgG. Fourteen proteins were screened for use in a diagnostic test but none possessed enough sensitivity and specificity to be useful. Six of the 9 proteins selected for the possibility that they may be immunomodulatory were not recognized by antibodies in patient serum.

**Conclusions:**

Thirty-three proteins that bound IgM and/or IgG from the serum of patients with ordinary scabies were identified. None of the 14 tested were useful for inclusion in a diagnostic test. The identities of 16 proteins that are not recognized as antigens by infected patients were also determined. These could be among the molecules that are responsible for this mite’s ability to modulate its host’s innate and adaptive immune responses.

## Author summary

Scabies, caused by the mite, *Sarcoptes scabiei*, infects millions of humans, and many wild and domestic mammals. Scabies mites burrow in the lower stratum corneum of the epidermis of the skin and are the source of substances that are antigenic or modulate aspects of the protective response of the host. Ordinary scabies is a difficult disease to diagnose. We identified 50 different proteins produced by *S*. *scabiei*, 33 of which bound IgM and/or IgG from the serum of patients with ordinary scabies. A set of 23 recombinant proteins were produced and screened for use in a diagnostic test but none possessed enough sensitivity and specificity to warrant further consideration although some could be among the molecules that are responsible for this mite’s ability to modulate its host’s innate and adaptive immune responses.

## Introduction

Scabies is a worldwide disease that affects millions of humans, other species of primates, and many wild and domestic mammals. It is caused by the itch mite, *Sarcoptes scabiei*, that burrows in the lower stratum corneum of the epidermis of the skin. Scabies mites are the source of substances that modulate certain aspects of the inflammatory innate and adaptive immune response of the host allowing it to evade detection by the host until it is able to establish a thriving population [1–12]. Ordinary scabies is a difficult disease to diagnose and there are no diagnostic blood tests with adequate sensitivity and specificity available to identify patients early in the course of an infection [13].

The goal of this project was to identify *S*. *scabiei* proteins that (1) may be candidate antigens for use in a diagnostic test or (2) may be among those used by the mite to modulate the host’s protective responses.

## Materials and methods

### Ethics statement

Serum from patients with confirmed ordinary scabies was collected under Human Subjects Protocol (HSP) #0205 as approved by the Wright State University Institutional Review Board (IRB). All patients were adults and all provided written informed consent. Negative control sera were previously provided to us without personal identifiers under protocol SC #2714 approved as EXEMPT under CFR 46.101(b)(4) by the Wright State University IRB.

### *S*. *scabiei* mite extract

An aqueous extract of *Sarcoptes scabiei* var. *canis* was prepared by homogenizing mites in endotoxin-free water as previously described [14]. Following two 24-hr extractions, the supernatants were collected by centrifugation, sterile-filtered (0.22 μm) into sterile vials and stored at 4°C. The protein content of this and all other samples was determined using the method of Bradford with bovine serum albumin (BSA) as standard [15].

### Protein separation

Unless otherwise noted, the materials used for protein separation and analysis were obtained from Bio-Rad Laboratories, Inc., Hercules, CA.

Proteins in the *S*. *scabiei* extract (40 mL containing 175 mg protein) were concentrated using preparative isoelectric focusing (IEF) as previously described [16] using a Bio-Rad Rotofor apparatus with ampholytes of pH 3–10 (BioLyte 3/10, 2% wt/vol final) and 5% glycerol. Focusing at 5°C for 5 hr at 12 W yielded 20 fractions with pH 1.6–13. Fractions 4–15 (pH 4–8 containing ~ 120 mg protein) were recombined and subjected to a second IEF separation. Fraction 14 had the highest protein concentration (2.2 mg/mL) and a pH of 5.0 and was selected for further study.

Two-dimensional (2D) gel electrophoresis was performed as previously described [14]. An aliquot of Fraction 14 was prepared using the ReadyPrep 2-D Cleanup Kit and the resulting protein sample was extracted into ReadyPrep Rehydration/Sample Buffer. Two identical samples, each containing ~200 μg of protein, were loaded onto 11 cm ReadyStrip pH 5–8 IPG strips using overnight passive rehydration. Second dimension separation was carried out using Criterion TGX Any kD precast gels as before. At the conclusion of the electrophoretic separation, one gel was stained with GelCode Blue Stain Reagent (Thermo Scientific, Rockford, IL). The other gel was prepared for electrophoretic transfer.

### Electrophoretic transfer and immunoblotting

Following 2D separation, the proteins on the second gel were transferred to an “Immun-Blot PVDF Membrane for Protein Blotting” using condition as previously described [17]. PBST, composed of Dulbecco’s Phosphate Buffered Saline + 1% Tween 20, was used as wash. BPBST (PBST+ 1% BSA + 1% normal goat serum) was used to block the membranes and for antibody dilutions except as noted.

A pool of serum from patients with confirmed ordinary scabies was prepared by combining equal volumes of 5 individual serum samples [18]. The serum pool was diluted 1/60 and used to probe the blot for 2 hrs. For IgM binding, the blot was probed for 1 hr in biotinylated-Goat anti-Human IgM at 1/5000 and 1hr in streptavidin-Alkaline Phosphatase at 1/5000 (both from Southern Biotechnology Associates, Birmingham, AL). Tris-buffered saline (TBS) replaced PBS in wash and diluent prior to the Alkaline Phosphatase step. The blot was developed using AP Blue Membrane Substrate (Sigma-Aldrich, St. Louis, MO) yielding blue spots where IgM bound. The blot was imaged and subsequently re-probed for IgG binding using biotinylated-Goat anti-Human IgG at 1/5000 and streptavidin-Horseradish Peroxidase at 1/5000 (Southern Biotechnology Associates). IgG binding proteins were stained reddish-brown using the substrate of Young [19]. Proteins that bound both IgM and IgG appeared purplish on the finished blot.

### Stained spot selection and protein identification

Both the stained gel and probed immunoblot were imaged and the images were overlaid with a 1,000-cell grid (25 row x 40 cells/row) as described before [14]. This allowed each stained protein spot on the gel and on the corresponding blot to be assigned a unique “spot number” identifier.

Ninety-seven blue-stained spots were excised from the gel using a 1-mm spot picker, collected into labeled LoBind tubes (Eppendorf, Westbury, NY) and frozen. Samples were shipped to Applied Biomics (Hayward, CA) for trypsin digestion and sequencing by mass spectrometry. Proteins were identified by MASCOT (Matrix Science, London, UK) search of the National Center for Biotechnology Information non-redundant database (NCBInr) with taxonomy restricted to “*Sarcoptes scabiei*”. This database contains the complete genome and predicted proteome for *S*. *scabiei* var. *canis* [20].

### Recombinant protein expression and purification

Gene sequences for selected proteins were synthesized by GenScript (Piscataway, NJ) with the open reading frame being codon-optimized for expression in *E*. *coli*. Additional modifications to the open reading frame were made to eliminate any internal BamHI, HindIII, and KpnI restriction sites. The termini of each gene contained in-frame 5' BamHI and 3' HindIII restriction sites for cloning into the pET-45b(+) expression vector. Expression vectors were transformed into *E*. *coli* Rosetta(DE3) competent cells (EMD Millipore, Billerica, MA). Transformants were selected on ampicillin-containing solid media plates, and 3-mL overnight liquid cultures were generated from five separate single colonies. The overnight cultures were incubated in liquid LB media that included ampicillin. All five cultures were then combined the following morning and subcultured into 500 mL of LB media without ampicillin for 3 hrs, followed by induction of protein expression by the addition of 1 mM (final concentration) IPTG for three hours. All liquid cultures were maintained in a MaxQ 4000 orbital shaking incubator (Thermo, Waltham, MA) shaking at 250 rpm and held at 32°C. Cells were harvested by centrifugation at 5000 x *g* for 20 min. Cell pellets were stored at -80°C until protein purification. Frozen cell pellets were resuspended in 10 mL of ice cold 1x Tris Buffered Saline (25 mM Tris, 150 mM NaCl, pH 7.2) containing Pierce Protease Inhibitor without EDTA (Thermo, Waltham, MA). Resuspended cells were disrupted by sonication on ice using 10 pulses of 30 sec on, 30 sec off with a 4710 series ultrasonic homogenizer (Cole Parmer, Vernon Hills, IL) set at 40% amplitude. Cellular debris was pelleted by centrifugation, and the supernatant was filtered through a 0.4 μm syringe filter. His-tagged proteins were then purified by column purification on Pierce His Pur Cobalt chromatography columns (Thermo) according to the manufacturer's recommendations and using a final elution volume of 3 mL. Purified proteins were quantified and analyzed as follows.

### Immunoscreening of recombinant proteins

All recombinant proteins were subjected to an initial immunoblot screening. Aliquots of purified proteins (3–10 μg) were loaded onto the single prep-well of Mini-Protean TGX Any kD Gels and electrophoresis was carried out at 200 V as recommended by the manufacturer (BioRad) and as described previously [21, 22]. Separated proteins were then transferred to PVDF membranes that were blocked as described above.

Ten pools of serum from patients with confirmed scabies infestations were prepared based on prior assessment [13, 18]. A pool of serum from healthy control subjects was included as a negative control. All proteins were tested with these sera. Eight proteins of interest were also screened with serum from 30 individual patients with ordinary scabies and 10 uninfested controls [13, 18].

Blots were loaded into a mini-slot blot apparatus (Mini-Protean Multiscreen, BioRad) [21, 22] and probed for 2 hrs with the sera as described above. After removal from the slot blot apparatus, blots were sequentially developed for IgM and IgG binding as described above.

The purity of all individual proteins was also determined using electrophoresis on Mini-Protean TGX Any kD Gels run as above and stained with GelCode Blue.

## Results

Our previous analysis revealed that most of the soluble proteins present in an aqueous extract of scabies mites had isoelectric points (pIs) in the range of pH 5–8 [14]. In the present analysis, we used preparative IEF to concentrate proteins with pIs in this vicinity and then used IPG strips of pH 5–8 for final separation.

**Fig 1:**
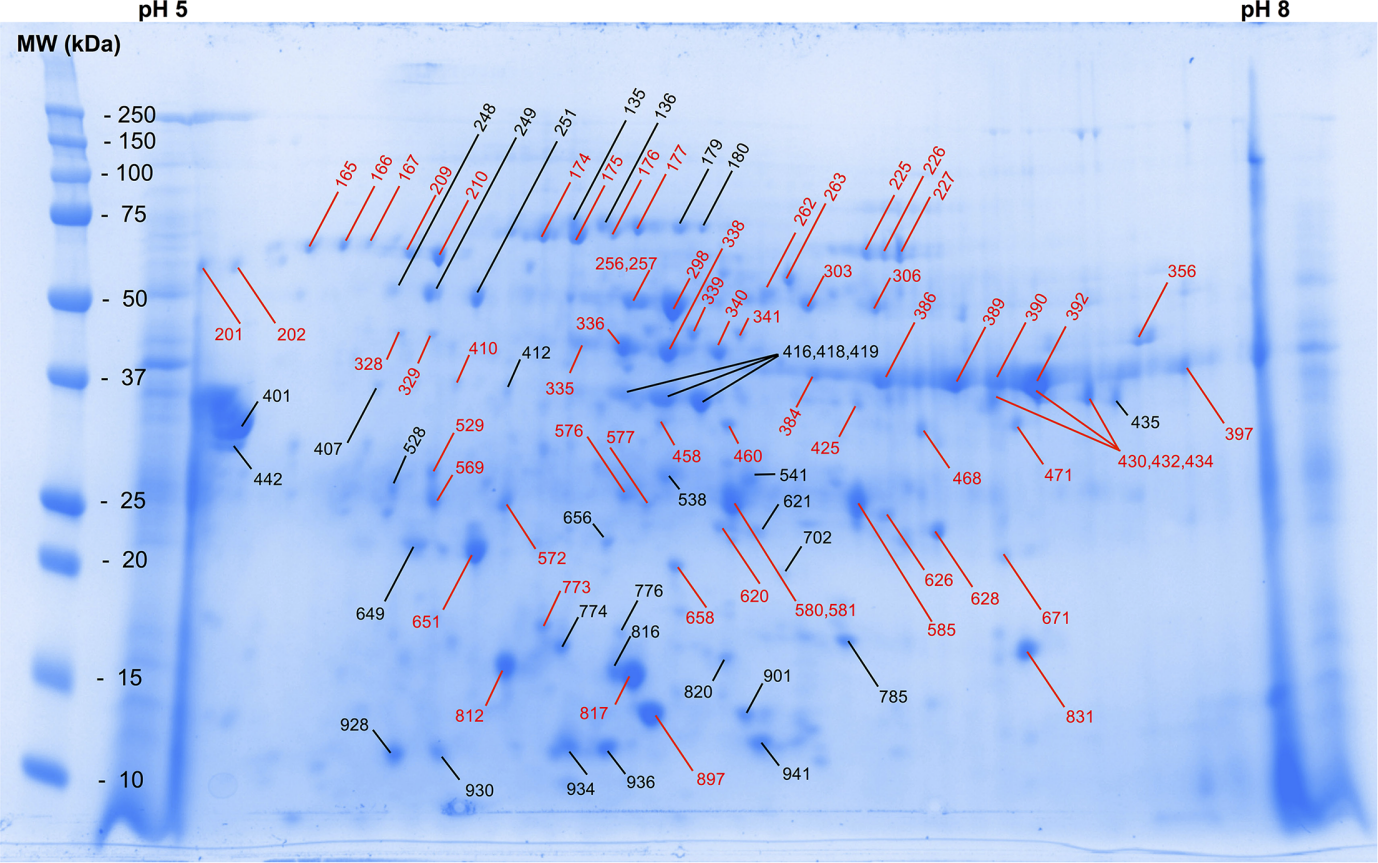
GelCode Coomassie blue stained 2-dimensional electrophoresis gel used to separate proteins of *S*. *scabiei*. Numbers in black denote those identified proteins observed only on the stained gel. Numbers in red indicate identified proteins that also bound antibody (immunoblot shown in Fig 2) from the serum of patients with ordinary scabies.

**Fig 2:**
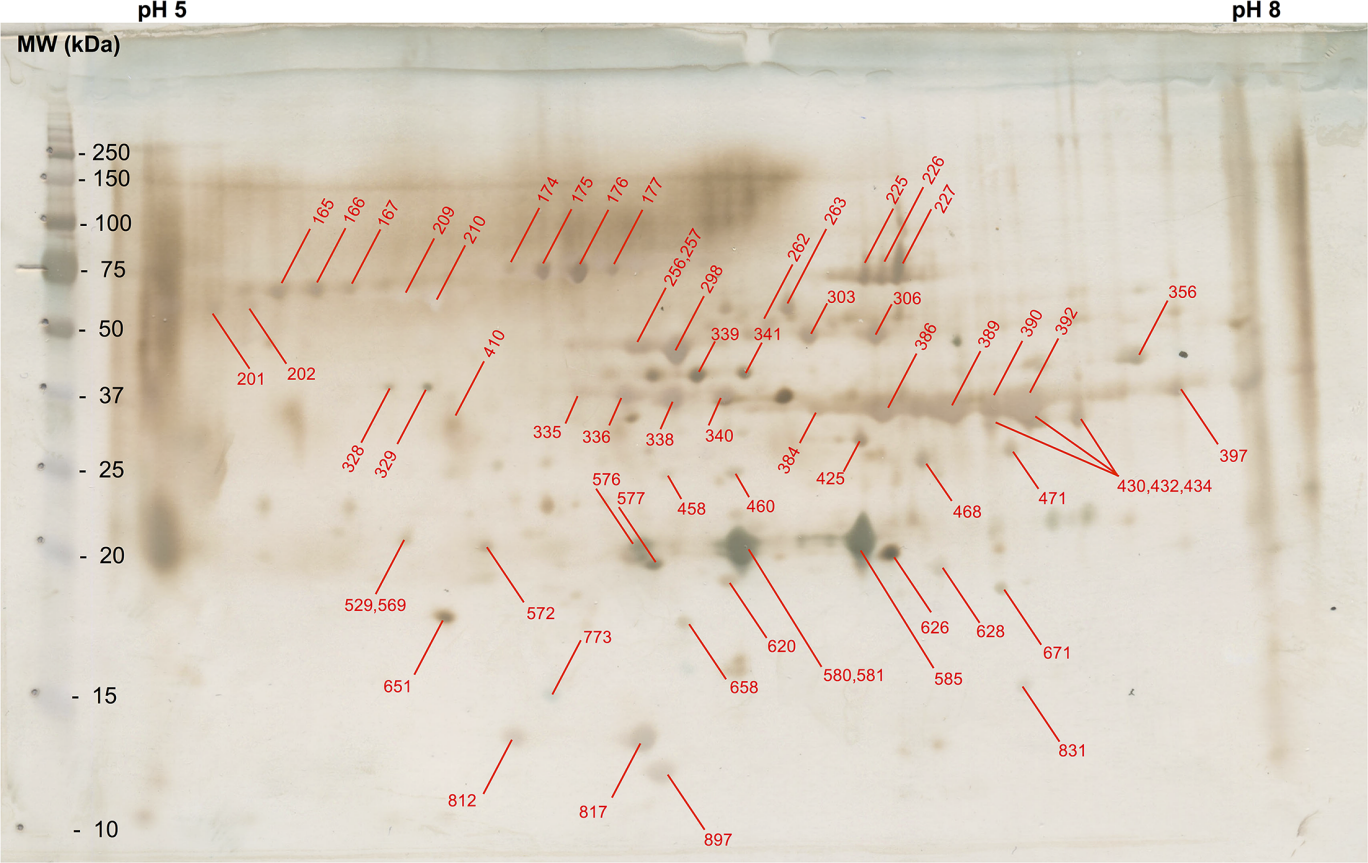
Immunoblot of 2-dimensional electrophoresis gel used to separate proteins of *S*. *scabiei*. Numbers in red indicate identified proteins that bound antibody from the serum of patients with ordinary scabies.

Ninety-seven protein-containing spots were excised from the GelCode Coomassie blue stained gel and were submitted for sequence analysis (Fig 1). All 97 spots were identified as containing one or more proteins of *S*. *scabiei* var. *canis* (Table 1). There were a total of 50 different *S*. *scabiei* proteins identified and 34 of these had not been previously reported (Fig 1, Table 1). The proteins from an identical gel were transferred to a PVDF membrane that was probed using a pool of sera from 5 patients with confirmed ordinary scabies infections that had previously been determined to have high levels of circulating antibodies that recognized antigens in *S*. *scabiei* extracts [13, 18]. Of the 97 protein-containing spots, one bound only IgM, 32 bound only IgG and 29 bound both IgM and IgG (Fig 2, Table 1). No antibody bound to 33 of the spots.

**Table 1:** Identification of the *S*. *scabiei* proteins in the 97 spots excised from the GelCode Blue stained 2-D gel (Fig 1) and their antibody binding profiles (Fig 2).

				MW		pI		
Spot #	New	Accession #	Protein Identification	Pred	Gel	Pred	Gel	Ab Binding
135		KPM11560	Sar s 28 (heat shock protein 70-like protein 8)	62,302	86,015	5.49	6.08	None
136		KPM11560	Sar s 28 (heat shock protein 70-like protein 8)	62,302	85,976	5.49	6.15	None
165	✔	KPM02263	Vitellogenin-like protein	220,647	70,206	6.03	5.38	IgM + IgG
166	✔	KPM02263	Vitellogenin-like protein	220,647	69,195	6.03	5.47	IgM + IgG
167	✔	KPM02263	Vitellogenin-like protein	220,647	70,799	6.03	5.53	IgM + IgG
174		KPM10172	Sar s 28 (heat shock protein 70-like protein 6)	77,075	71,005	6.50	6.01	IgG only
175		KPM10172	Sar s 28 (heat shock protein 70-like protein 6)	77,075	72,738	6.50	6.07	IgG only
176		KPM10172	Sar s 28 (heat shock protein 70-like protein 6)	77,075	74,713	6.50	6.15	IgG only
177		KPM11560	Sar s 28 (heat shock protein 70-like protein 8)	62,302	75,188	5.49	6.22	IgG only
179		KPM11560	Sar s 28 (heat shock protein 70-like protein 8)	62,302	74,328	5.49	6.35	None
180	✔	KPM08931	Elongation factor G, mitochondrial-like protein	84,242	73,932	7.53	6.42	None
201	✔	KPM02263	Vitellogenin-like protein	220,647	60,438	6.03	5.10	IgG only
202	✔	KPM02263	Vitellogenin-like protein	220,647	60,675	6.03	5.19	IgG only
209		KPM06690	60 kDa Heat shock protein, mitochondrial-like protein	62,505	61,556	5.77	5.67	Neg stain
210		KPM06690	60 kDa Heat shock protein, mitochondrial-like protein	62,505	62,973	5.77	5.72	Neg stain
225	✔	KPM07637	Actin-interacting protein 1-like protein	64,294	62,437	6.07	6.78	IgG only
226	✔	KPM07637	Actin-interacting protein 1-like protein	64,294	62,437	6.07	6.78	IgG only
227	✔	KPM07637	Actin-interacting protein 1-like protein	64,294	63,960	6.07	6.90	IgG only
248	✔	KPM02263	Vitellogenin-like protein	220,647	53,200	6.03	5.60	None
249	✔	KPM02263	Vitellogenin-like protein	220,647	52,287	6.03	5.68	None
251	✔	KPM02263	Vitellogenin-like protein	220,647	51,639	6.03	5.81	None
256		KPM02829	Enolase-like protein	47,320	53,202	5.75	6.16	IgG only
257		KPM02829	Enolase-like protein	47,320	53,321	5.75	6.22	IgG only
262	✔	KPM04850	Alpha-aminoadipic semialdehyde dehydrogenase-like protein	59,780	53,297	6.77	6.56	IgG only
263	✔	KPL93612	Pyruvate kinase-like protein	56,757	54,597	6.03	6.63	IgG only
298		KPM02829	Enolase-like protein	47,320	47,163	5.75	6.30	IgM + IgG
303	✔	KPM04598	Hypothetical protein QR98_0030480	53,449	48,048	6.13	6.65	IgM + IgG
306	✔	KPM04598	Hypothetical protein QR98_0030480	53,449	48,011	6.13	6.84	IgM + IgG
328		ACC85688	Actin	41,570	42,022	5.22	5.61	IgM + IgG
329		KPM11937	Actin-like protein 6	16,941	42,138	4.66	5.68	IgM + IgG
335	✔	KPM03560	Sar s 27 allergen (serpin-like protein 9)	46,965	40,933	5.68	6.08	IgG only
336		KPM02829	Enolase-like protein	47,320	40,307	5.75	6.17	IgG only
338		KPM02829	Enolase-like protein	47,320	40,313	5.75	6.30	IgG only
339	✔	KPM09149	Fumarylacetoacetase-like protein	47,804	41,329	5.77	6.36	IgM + IgG
340		KPM02829	Enolase-like protein	47,320	40,148	5.75	6.43	IgG only
341	✔	KPM09149	Fumarylacetoacetase-like protein	47,804	42,081	5.77	6.49	IgM + IgG
356	✔	KPM04355	Isocitrate dehydrogenase [NADP] cytoplasmic-like protein	46,226	41,590	6.80	7.53	IgM + IgG
384*		KPM02376	Sar s 20 allergen (arginine kinase like 1)	33,971	36,036	6.29	6.70	IgG only
384*	✔	KPM11752	Hypothetical protein QR98_0103270	39,594		8.07		IgG only
386*		KPM02376	Sar s 20 allergen (arginine kinase like 1)	33,971	36,128	6.29	6.85	IgG only
386*	✔	KPM11752	Hypothetical protein QR98_0103270	39,594		8.07		IgG only
389*		KPM02376	Sar s 20 allergen (arginine kinase like 1)	33,971	36,087	6.29	7.05	IgG only
389*	✔	KPM11752	Hypothetical protein QR98_0103270	39,594		8.07		IgG only
390*		KPM02376	Sar s 20 allergen (arginine kinase like 1)	33,971	36,618	6.29	7.13	IgG only
390*	✔	KPM11752	Hypothetical protein QR98_0103270	39,594		8.07		IgG only
392		KPM02376	Sar s 20 allergen (arginine kinase like 1)	33,971	36,162	6.29	7.25	IgG only
397	✔	KPM11752	Hypothetical protein QR98_0103270	39,594	36,692	8.07	7.62	IgG only
401		AFH08744	Tropomyosin	32,907	32,450	4.75	5.12	None
407		KPM02829	Enolase-like protein	47,320	33,449	5.75	5.54	None
410	✔	KPM05552	Sar s 32 allergen (inorganic pyrophosphatase-like protein)	34,225	33,805	5.40	5.75	IgG only
412	✔	KPM02263	Vitellogenin-like protein	220,647	33,131	6.03	5.88	None
416	✔	KPM08991	Hypothetical protein QR98_0075200	9,170	32,823	6.73	6.17	None
418	✔	KPM08991	Hypothetical protein QR98_0075200	9,170	32,135	6.73	6.29	None
419	✔	KPM06764	Mediator of RNA polymerase II transcription subunit 8-like protein	26,845	32,255	7.79	6.37	None
425	✔	KPM03187	Ester hydrolase C11orf54-like protein	36,806	32,243	6.11	6.77	IgM + IgG
430	✔	KPM05576	Glyceraldehyde-3-phosphate dehydrogenase 2-like protein	36,680	33,062	6.67	7.12	IgM + IgG
432		KPM02376	Sar s 20 allergen (arginine kinase like 1)	33,971	33,121	6.29	7.25	IgG only
434	✔	KPM05576	Glyceraldehyde-3-phosphate dehydrogenase 2-like protein	36,680	33,121	6.67	7.38	IgM + IgG
435	✔	KPM02263	Vitellogenin-like protein	220,647	32,425	6.03	7.46	None
442		AFH08744	Tropomyosin	32,907	29,803	4.75	5.20	None
458	✔	KPM10460	Glyoxalase domain-containing protein 4-like protein	30,916	29,727	5.23	6.29	IgG only
460	✔	KPM10460	Glyoxalase domain-containing protein 4-like protein	30,916	29,561	5.23	6.43	IgG only
468		KPM04864	Malate dehydrogenase, cytoplasmic-like protein	34,948	28,983	6.07	6.97	IgM + IgG
471		KPM04864	Malate dehydrogenase, cytoplasmic-like protein	34,948	29,124	6.07	7.19	IgM + IgG
528	✔	KPM11752	Hypothetical protein QR98_0103270	39,594	23,785	8.07	5.59	None
529		KPM11560	Sar s 28 (heat shock protein 70-like protein 8)	62,302	24,258	5.49	5.68	IgG only
538	✔	KPM02928	Citrate synthase 1, mitochondrial-like protein	51,707	24,123	7.75	6.30	None
541	✔	KPM04580	Sar s 16 allergen (gelsolin-like protein)	54,930	23,992	6.08	6.50	None
569		KPM11560	Sar s 28 (heat shock protein 70-like protein 8)	62,302	22,281	5.49	5.68	IgG only
572		KPM11560	Sar s 28 (heat shock protein 70-like protein 8)	62,302	22,111	5.49	5.88	IgG only
576*	✔	KPM11822	Dehydrogenase/reductase SDR family member 2-like protein	58,241	22,320	8.09	6.16	IgM + IgG
576*	✔	KPM03144	Haloacid dehalogenase-like hydrolase domain-containing protein	31,713		5.63		IgM + IgG
577	✔	KPM02528	Proteasome subunit beta type-4-like protein	27,132	22,360	5.79	6.21	IgM + IgG
580	✔	KPM11822	Dehydrogenase/reductase SDR family member 2-like protein	58,241	22,082	8.09	6.44	IgM + IgG
581	✔	KPM11822	Dehydrogenase/reductase SDR family member 2-like protein	58,241	21,974	8.09	6.49	IgM + IgG
585	✔	KPM11822	Dehydrogenase/reductase SDR family member 2-like protein	58,241	22,073	8.09	6.79	IgM + IgG
620	✔	KPM02662	Proteasome subunit alpha type-2-like protein	47,963	20,507	10.13	6.43	IgG only
621		ACX33880	Glutathione S-transferase delta class 3, partial	21,192	20,680	6.13	6.49	None
626*	✔	KPM09477	Proteasome subunit alpha type-6-like protein	26,986	20,751	6.24	6.85	IgM + IgG
626*	✔	KPM10536	Short-chain alcohol dehydrogenase-like protein	26,299		5.57		IgM + IgG
628		KPM10468	Sar s 25 allergen (triosephosphate isomerase-like protein)	26,678	20,128	6.13	6.99	IgM + IgG
649	✔	KPM04725	Sar s 30 allergen (ferritin-like protein 3)	21,807	19,090	5.19	5.66	None
651	✔	KPM04725	Sar s 30 allergen (ferritin-like protein 3)	21,807	18,916	5.19	5.80	IgG only
656	✔	KPM04725	Sar s 30 allergen (ferritin-like protein 3)	21,807	19,261	5.19	6.15	None
658	✔	KPM03215	Phosphatidylethanolamine-binding protein-like protein F40A3.3-like protein	20,465	18,433	5.87	6.30	IgM + IgG
671	✔	KPM03215	Phosphatidylethanolamine-binding protein-like protein F40A3.3-like protein	20,465	18,655	5.87	7.17	IgM + IgG
702	✔	KPM03215	Phosphatidylethanolamine-binding protein-like protein F40A3.3-like protein	20,465	17,609	5.87	6.58	None
773	✔	KPM03156	14-3-3 protein-like protein 1	28,027	15,193	4.79	5.95	IgM only
774	✔	KPM06865	Disulfide-isomerase A3-like protein	59,046	15,064	5.78	6.02	None
776*		KPL97138	Stress-induced-phosphoprotein 1-like protein	37,044		7.53		None
776*	✔	KPM02263	Vitellogenin-like protein	220,647	15,071	6.03	6.16	None
785	✔	KPM11739	Superoxide dismutase [Cu-Zn]-like protein	16,109	14,874	6.02	6.76	None
812	✔	KPM08623	Sar s 31 allergen (cofilin-like protein)	16,821	13,931	5.95	5.88	IgM + IgG
816	✔	KPM02263	Vitellogenin-like protein	220,647	13,965	6.03	6.16	None
817	✔	KPM08623	Sar s 31 allergen (cofilin-like protein)	16,821	13,923	5.95	6.22	IgM + IgG
820	✔	KPM02263	Vitellogenin-like protein	220,647	14,107	6.03	6.45	None
831		KPM06968	Nucleoside diphosphate kinase B-like protein	14,100	14,241	9.88	7.21	IgM + IgG
897	✔	KPM07763	Sar s 13 allergen (lipocalin-like protein)	14,867	12,328	6.37	6.24	IgM + IgG
901	✔	KPM07609	CUB domain containing protein 4	122,775	12,301	5.67	6.50	None
928		KPM09467	Thioredoxin-like protein 2	11,704	11,508	5.06	5.60	None
930	✔	KPM04598	Hypothetical protein QR98_0030480	53,449	11,587	6.13	5.73	None
934	✔	KPL94049	Muscular protein 20-like protein	15,837	11,581	6.73	6.02	None
936*	✔	KPM03769	Hypothetical protein QR98_0022030	18,008	11,514	4.81	6.15	None
936*	✔	KPL94049	Muscular protein 20-like protein	15,837		6.73		None
941	✔	KPL94049	Muscular protein 20-like protein	15,837	11,560	6.73	6.51	None

Proteins are denoted by spot numbers that correspond to those shown in Figs 1 and 2.

* indicates that the spot contained a mixture of proteins. ✔ denotes proteins that were not previously identified [14]. Molecular weights (MW) and isoelectric points (pI) predicted for each protein and determined from the gel are given. Immunoblot binding of IgM only, IgG only or both IgM and IgG is indicated. Some proteins did not bind antibody (None) and two proteins appeared negatively stained (white spots) on the immunoblot.

We previously postulated that a diagnostic test for scabies would require identifying a set of antigens that selectively bind antibody (especially IgM) from the serum of patients suspected of being infected with scabies mites [13]. Based on the antibody-binding profiles of the proteins identified on the 2D gel and blot, we selected 14 proteins for further study as diagnostic antigen candidates (Table 2). We also selected 9 additional proteins from the > 150 previously-identified proteins that could be among the molecules that are responsible for this mite’s ability to modulate its host’s immune responses (Table 2) [12, 14]. The genes coding for these 23 proteins were deduced from the *S*. *scabiei* var. *canis* genome [20], chemically synthesized, and cloned into *E*. *coli*. The recombinant proteins were expressed and partially purified before being subjected to immunoblot screening. GelCode Coomassie blue stained gels showed that the purity of the recombinant proteins ranged from 10% to 95%.

**Table 2:** First round screening of recombinant *S*. *scabiei* proteins for their ability to bind IgM and/or IgG from the serum of patients with ordinary scabies.

Accession #	Protein Identification	IgM	IgG	IgM -/+ IgG
	**Diagnostic antigen candidates**			
KPM03156	14-3-3 protein-like protein 1	7/10	10/10	10/10
KPM09740	2,3-bisphosphoglycerate-independent phosphoglycerate mutase-like protein	-	-	-
KPM11822	Dehydrogenase/reductase SDR family member 2-like protein	-	1/10	1/10
KPM02829	Enolase-like protein	10/10	10/10	10/10
KPM03144	Haloacid dehalogenase-like hydrolase domain-containing protein	-	2/10	2/10
KPM04864	Malate dehydrogenase, cytoplasmic-like protein	-	-	-
KPM03215	Phosphatidylethanolamine-binding protein-like protein F40A3.3-like protein	4/10	5/10	5/10
KPM09477	Proteasome subunit alpha type-6-like protein	-	-	-
KPM07763	Sar s 13 allergen (lipocalin-like protein)	3/10	10/10	10/10
KPM10468	Sar s 25 allergen (triosephosphate isomerase-like protein)	10/10	10/10	10/10
KPM08623	Sar s 31 allergen (cofilin-like protein)	5/10	10/10	10/10
KPM10536	Short-chain alcohol dehydrogenase-like protein	-	-	-
KPM11752	Hypothetical protein QR98_0103270	10/10	10/10	10/10
KPM04725	Sar s 30 allergen (ferritin-like protein 3)	3/10	10/10	10/10
	**Immunomodulatory molecule candidates**			
KPM11739	Superoxide dismutase [Cu-Zn]-like protein	-	-	-
KPM07532	Calmodulin-like	-	2/10	2/10
KPM04170	Calreticulin-like	1/10	-	1/10
KPM09372	Cystatin-C-like protein	-	-	-
KPM09373	Cystatin-like protein	-	-	-
KPM09123	Hypothetical protein QR98_0076540	-	-	-
KPM07951	Mannan-binding lectin serine protease 1-like	-	-	-
KPM05304	Matrix metalloproteinase-like	-	-	-
KPM02741	Membrane metallo-endopeptidase-like 1-like	1/10	2/10	2/10

Each protein was tested with 10 pools of serum from patients with ordinary scabies. The number of pools out of 10 that had specific antibody isotypes directed at each protein is shown.

For the first round of immunoscreening, ten pools of serum from patients with confirmed scabies infections were prepared based on prior screening [13, 18]. Another pool of sera from uninfected subjects was also prepared to serve as a negative control. Each of the 23 proteins was then screened with these 11 serum pools by slot blot. Eight of the 14 proteins that were selected as diagnostic antigen candidates were recognized by antibodies in ≥ 50% of the test serum pools (Table 2). None of the candidate immunomodulatory proteins bound antibodies in more than 20% of the test sera.

The 8 most promising diagnostic antigen candidates were subjected to a second round of screening using the serum of 30 individual US patients with ordinary scabies and 10 uninfected controls [13, 18]. One protein (KPM11752), a hypothetical protein that appears unique to scabies mites, was recognized by antibodies present in the serum of all scabies patients and all control subjects (Table 3). Three other proteins (KPM03215, KPM07763 and KPM10468) bound antibodies present in the serum of 40–67% of the scabies patients but they were also recognized by 10–40% of the control sera. Two of these are homologs of the Group 13 and 25 dust mite allergens. The remaining candidate proteins were recognized by ≤ 30% of the serum from the scabies patients.

**Table 3:** Second round screening of recombinant *S*. *scabiei* proteins for their ability to bind IgM and/or IgG from the serum of patients with ordinary scabies and from uninfected control subjects.

			Patients		Controls
Accession #	Protein Identification	IgM	IgG	IgM -/+ IgG	IgM -/+ IgG
KPM03156	14-3-3 protein-like protein 1	4/30	7/30	7/30	2/10
KPM02829	Enolase-like protein	0/30	3/30	3/30	0/10
KPM03215	Phosphatidylethanolamine-binding protein-like protein F40A3.3-like protein	12/30	12/30	12/30	1/10
KPM07763	Sar s 13 allergen (lipocalin-like protein)	11/30	15/30	20/30	4/10
KPM10468	Sar s 25 allergen (triosephosphate isomerase-like protein)	6/30	7/30	13/30	3/10
KPM08623	Sar s 31 allergen (cofilin-like protein)	9/30	9/30	9/30	3/10
KPM11752	Hypothetical protein QR98_0103270	30/30	30/30	30/30	10/10
KPM04725	Sar s 30 allergen (ferritin-like protein 3)	3/30	3/30	5/30	0/10

Each protein was tested with serum from 30 individual patients with ordinary scabies and from 10 uninfected control subjects. The number of individual sera out of 30 that had specific antibody isotypes directed at each protein is shown.

## Discussion

This research builds on previous proteomic work by identifying 50 different proteins produced by *S*. *scabiei*, 34 of which were not identified previously [14]. We determined that 66% of the protein-containing spots were recognized by IgM and/or IgG that is circulating in the serum of patients with ordinary scabies at the time of initial diagnosis and selected 14 of these for screening as candidates for use in a diagnostic test for scabies. Additionally, we identified 33 protein-containing spots, representing 16 different proteins, that were isolated from a Coomassie blue stained gel that did not bind patient antibody. Included among this set of proteins may be molecules that participate in the parasite’s immune evasion mechanisms and are responsible for modulating the host’s immune responses [1–12].

Unfortunately none of the 14 proteins selected as potential diagnostic antigens shows enough promise to warrant further study. Only two proteins (KPM07763 and KPM11752) had sensitivities of ≥ 67% but neither offered a specificity of > 40% (Table 3). An additional 19 different antibody-binding proteins were identified on the 2D immunoblot and these are also potential candidates for use as diagnostic antigens (Table 1, Fig 2). It is possible that screening of these proteins could yield candidates promising better sensitivity and specificity than those reported here.

Perhaps more interesting are the data for the 9 proteins selected for the possibility that they may be immunomodulatory. All were identified in a previous study [14] or were predicted from the genome [20] and none were detected on the 2D immunoblot (Fig 2). Among these were calmodulin-, calreticulin- and cystatin-like proteins, all of which have been shown to be produced by other parasites and to possess immunomodulatory properties [23, 24]. For a protein to be effective in assisting the mite to evade the host’s immune response it would likely also be able to escape detection by the adaptive immune system and would not elicit an antibody response. As would be expected, 3 of the 9 proteins tested were recognized by antibodies in the serum of ≤ 20% of the scabietic patients while the other 6 did not bind any antibody. A logical next step would be to test these proteins for their immunomodulatory properties, although this was beyond the scope of the present study.

### Conclusions

Thirty-three proteins that bound IgM and/or IgG from the serum of patients with ordinary scabies were identified. Although none of the 14 tested are useful for inclusion in a diagnostic test, the identity of 19 other candidates is provided. The identity of 16 proteins that are not recognized as antigens by infected patients was also determined. These could be among the molecules that are responsible for this mite’s ability to modulate its host’s innate and adaptive immune responses.
